# Coverage and rate analysis in the uplink of millimeter wave cellular networks with fractional power control

**DOI:** 10.1186/s13638-018-1208-0

**Published:** 2018-08-10

**Authors:** Oluwakayode Onireti, Ali Imran, Muhammad A. Imran

**Affiliations:** 1 School of Engineering, University of Glasgow, University Avenue, Glasgow, G12 8QQ UK; 2 School of Electrical and Computer Engineering, University of Oklahoma, Tulsa, OK USA

**Keywords:** 5G cellular network, Fractional power control, Millimeter wave, Stochastic geometry, Uplink

## Abstract

In this paper, using the concept of stochastic geometry, we present an analytical framework to evaluate the signal-to-interference-and-noise-ratio (SINR) coverage in the uplink of millimeter wave cellular networks. By using a distance-dependent line-of-sight (LOS) probability function, the location of LOS and non-LOS users are modeled as two independent non-homogeneous Poisson point processes, with each having a different pathloss exponent. The analysis takes account of per-user fractional power control (FPC), which couples the transmission of users based on location-dependent channel inversion. We consider the following scenarios in our analysis: (1) pathloss-based FPC (PL-FPC) which is performed using the measured pathloss and (2) distance-based FPC (D-FPC) which is performed using the measured distance. Using the developed framework, we derive expressions for the area spectral efficiency. Results suggest that in terms of SINR coverage, D-FPC outperforms PL-FPC scheme at high SINR where the future networks are expected to operate. It achieves equal or better area spectral efficiency compared with the PL-FPC scheme. Contrary to the conventional ultra-high frequency cellular networks, in both FPC schemes, the SINR coverage decreases as the cell density becomes greater than a threshold, while the area spectral efficiency experiences a slow growth region.

## Introduction

Increasing bandwidth by moving into the millimeter wave (mmWave) band has been identified as one of the primary approaches towards meeting the data rate requirement of the fifth generation (5G) cellular networks [1–3]. According to [2], the available spectrum for cellular communications at the mmWave band can be easily 200 times greater than the spectrum presently allocated for this purpose below the 3 GHz [2]. The mmWave band ranging from 30−300 GHz has already been considered for wireless services such as fixed access and personal area networking [4, 5]. However, such frequency bands have long been deemed unsuitable for cellular communications due to the large free space pathloss and poor penetration (i.e., blockage effect) through materials such as water and concrete. Only recently did survey measurements and capacity studies of mmWave technology reveal its promise for urban small cell deployments [1, 6–8]. In addition to the huge available bandwidth in the mmWave band, the smaller wavelength associated with the band allows for the use of more miniaturized antennas at the same physical area of the transmitter and receiver to provide array gain [2, 6]. With such large antenna array, the mmWave cellular system can apply beamforming at the transmit and receive sides to provide array gain which compensates for the near-field pathloss [9].

A major distinguishing factor in mmWave is the propagation environment. As a result of the blockage effect associated with mmWave, outdoor mmWave base stations (BSs) are more likely to serve outdoor users since mmWave signals suffer severe penetration losses [10]. Also, it has been revealed via the channel measurement in [1, 8] that blockages result in a significant difference between the line-of-sight (LOS) and non-line-of-sight (NLOS) pathloss characteristics. The measurement showed that mmWave signals propagate with pathloss exponent of 2 in LOS paths and a much higher pathloss exponent with additional shadowing in NLOS paths [1, 8]. Furthermore, the NLOS pathloss exponent tends to be more dependent on the scattering environment [11], with typical measured values ranging from 3.2 to 5.8 [1, 8].

### Related work

Recently, the use of stochastic geometry-based analysis was proposed to assess the capacity of conventional UHF cellular systems in [12–17]. Focusing on the downlink channel of the conventional UHF cellular networks, the authors in [12] modeled the BS location as a Poisson point process (PPP) on the plane and derived the signal-to-interference-and-noise-ratio (SINR) coverage probability and the average rate of a typical user. An extension of the stochastic model to the uplink channel of the conventional UHF cellular network, which is based on the dependence assumption where user and BS point processes are such that each BS serves a single user in a given resource block, is presented in [13]. The authors in [13] also included a per-user fractional power control (FPC) in their model. The results in [12] have also been extended to the multi-tier UHF cellular networks in [14–17] and for systems performance analysis in [18–20]. However, as a result of blockage and the different propagation model, the result obtained for the UHF networks are not applicable to the mmWave networks.

In order to analyze the system performance in mmWave cellular networks, a stochastic blockage model, where the blockage parameters are characterized by some random distributions, was presented for such networks in [21]. Also using the stochastic blockage process, authors in [11] proposed a framework to analyze the SINR and rate coverage probability of mmWave networks in the downlink channel while considering outdoor mmWave BSs and outdoor users. In [22], a more comprehensive analytic framework for mmWave cellular networks, which further incorporates self-backhauling but with a simplified blockage model, was presented.

### Contribution and organization

In this paper, we present a stochastic geometry framework for evaluating the SINR coverage in the uplink of mmWave cellular networks with per-user FPC. The aim of FPC is to minimize mobile (user) battery consumption and minimize interference to other cells. We consider two forms of FPC: (1) Pathloss-based FPC (PL-FPC), which is the conventional approach and is based on the measured pathloss and (2) distance-based FPC (D-FPC), which is based on the measured distance. In Section 2, we describe the methodology. In particular, we present the system model for mmWave networks and review expressions of the distribution of the distance between a typical user and its serving BS. We model the location of users and BSs as realizations of the PPP. Similar to [11], we introduce the blockage effect by modeling the probability that a link is LOS as a function of the link length. We then model the transmit power of the users based on both FPC schemes. Based on this modeling, it occurs that the random variables denoting this distance for each user (LOS or NLOS) are identically distributed but not independent in general. Hence, in Section 3, we first prove that this dependence is weak and can therefore be ignored for analytical tractability. Next, based on the independence assumption, we present a formal proof of the SINR coverage probability for both the pathloss- and distance-based FPCs. Afterwards, we derive a much-simplified expression for the noise-limited scenario. Next, using the developed framework, we derive the area spectral efficiency for the uplink of mmWave cellular networks. Numerical results and discussions are presented in Section 4. Results show the accuracy of our framework for a wide range of system parameters and that D-FPC outperforms the PL-FPC at high SINR threshold. Furthermore, contrary to UHF cellular networks, the area spectral efficiency in the mmWave cellular networks suffers a slow growth region as the BS density increases. Conclusions are finally drawn in Section 5.

## Methodology

### Network model

We consider the uplink of a mmWave cellular network and focus on the SINR coverage experienced by outdoor users served by outdoor BSs. The outdoor BSs are spatially distributed in $\mathbb {R}^{2}$ according to an independent homogeneous PPP with density *λ*. The user location (before association) are assumed to form a realization of homogeneous PPP with density *λ*_*u*_. Each BS serves a single user per channel which is randomly selected from all the users located in its Voronoi cell. Hence, the user PPP *λ*_*u*_ is thinned to obtain a point process *Φ*={*X*_*z*_}, where *X*_*z*_ is the location active outdoor user. As in [16, 18, 23], we assume that the active users also form PPP even after associating just one user per BS. Since we have one active user per cell, the density *ϕ* of the thinned PPP of active users is set to be equal to the BS density *λ*.

**Fig. 1:**
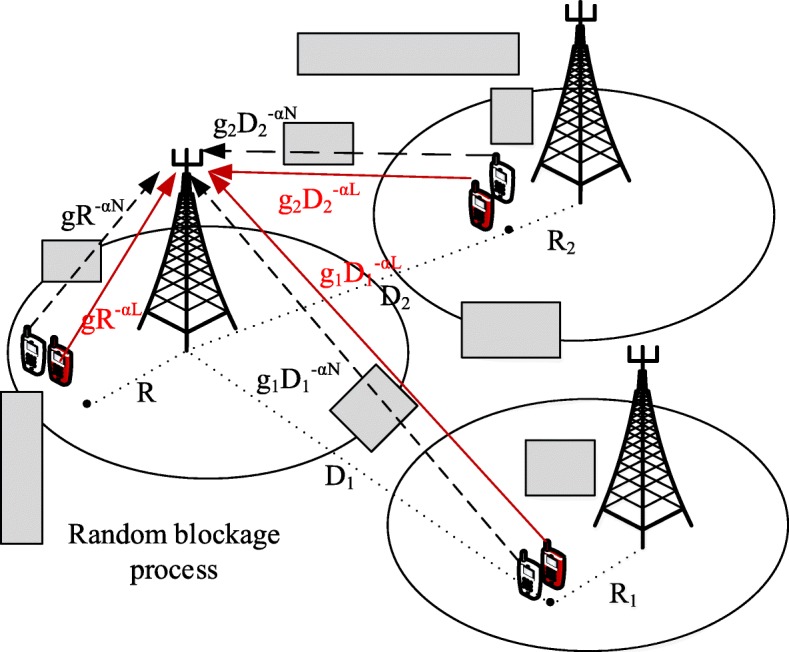
Visual representation of the uplink of mmWave cellular networks, focusing on the serving user and two interfering users in adjacent cells. Blockages are modeled as random process of rectangles as in [11]. White and red color marked users denotes the LOS and NLOS representation of the same user

The blocking effect is modeled according to [11], and we perform our analysis on a typical outdoor user whose connected BS is termed as the reference BS. An outdoor user can either be LOS or NLOS to the reference BS, as illustrated in Fig. 1. Let *Φ*_*L*_ be the point process of the LOS users, and *Φ*_*N*_ be the process of NLOS users. We define the LOS probability function *p*(*R*) as the probability that a link of length *R* is LOS. The NLOS probability of the link is 1−*p*(*R*). Different pathloss models are applied to the LOS and NLOS links. Hence, given a link that has length *R*, its pathloss gain *L*(*R*) can be computed as

1 $$ L(R)= \mathbb{I}\left(p(R)\right)C_{L}R^{-\alpha_{L}}+\left(1-\mathbb{I}\left(p(R)\right)\right)C_{N}R^{-\alpha_{N}},  $$

where $\mathbb {I}(r)$ is a Bernoulli random variable with parameter *r*, *C*_*L*_ and *C*_*N*_ are the intercepts on the LOS and NLOS pathloss expressions, and *α*_*L*_ and *α*_*N*_ are the LOS and NLOS pathloss exponents. The LOS probability function is modeled from a stochastic blockage model, where the blockage is modeled as a rectangle Boolean scheme. *p*(*R*)=*e*^−*βR*^, where *β* is a parameter determined by the average size and density of the blockages [21]. We assume that each user, either LOS or NLOS, associates with the BS that offers the maximum long-term averaged received power, i.e, the effect of fading is averaged out and hence ignored.

### Independent LOS probability

Without loss of accuracy, we ignore the correlation of the blockage effects between the links as demonstrated in [21] and assume that the LOS probabilities are independent between links. Consequently, the LOS user process *Φ*_*L*_ and the NLOS process *Φ*_*N*_ form two independent non-homogeneous PPPs with density functions *λp*(*R*) and *λ*(1−*p*(*R*)), respectively, where *R* is the Euclidean distance between a sender and receiver. Following the independence of the LOS probability, the distributions of the distance between the reference BS and, a LOS or NLOS typical user are given next.

#### Distribution of the distance *R*_*L*_ between the reference BS and a LOS typical user

Given that the typical user has a LOS association with the reference BS, the probability distribution function (PDF) of the distance *R*_*L*_ between the typical user and the reference BS can be expressed from [11] as

2 $$ {\begin{aligned} {}\mathcal{F}_{\!R_{L\!}}(r)\,=\,\frac{2\pi\!\lambda r e^{-\beta r}}{\mathcal{A}_{L}}\!\exp{\!\left(\!-2\pi\!\lambda\!\left(\!\frac{\left(\beta q_{_{l}}r^{v_{l}}\,+\,1\right)\!e^{-\beta q_{l}r^{v_{l}}}}{\beta^{2}}\,-\,\frac{\left(\beta r\,+\,1\right)e^{-\beta r}}{\beta^{2}}\,+\,\frac{q_{l}^{2}r^{2v_{l}}}{2}\!\right)\!\right)} \end{aligned}}  $$

where $q_{l}=\left (C_{N}/C_{L}\right)^{\frac {1}{\alpha _{N}}}, v_{l}=\alpha _{L}/\alpha _{N} $ and

3 $$ {{\begin{aligned} {}\mathcal{A}_{L}\,=\,2\pi\lambda\!\!\int_{0}^{\infty} \!\!{r e^{-\beta r}}\!\!\exp\!\left(\!\!-2\pi\!\lambda\!\left(\!\frac{\left(\beta q_{l}r^{v_{l}}\,+\,1\right)e^{-\beta q_{l}r^{v_{l}}}}{\beta^{2}}-\frac{\left(\beta r\,+\,1\right)e^{-\beta r}}{\beta^{2}}\,+\,\frac{q_{l}^{2}r^{2v_{l}}}{2}\!\right)\!\right)\!\mathrm{\!d}r \end{aligned}}}  $$

is the probability that the reference BS is connected to a LOS typical user.

#### Distribution of the distance *R*_*N*_ between the reference BS and a NLOS typical user

Given that the typical user has a NLOS association with the reference BS, the PDF of the distance *R*_*N*_ between the typical user and the reference BS can be expressed from [11] as

4 $$ {{\begin{aligned} {}\mathcal{F}_{\!R_{N}}\!(r)\,=\,\frac{2\pi\lambda r \left(\!1\!\,-\,e^{-\beta r}\right)}{\mathcal{A}_{N}}\exp\!\!\left(\!\!-2\pi\lambda\!\!\left(\!\frac{\left(\beta r\!\,+\,\!1\right)\!e^{-\beta r}}{\beta^{2}}\,-\,\frac{\left(\beta q_{n}r^{v_{n}}\!\,+\,\!1\right)\!e^{\,-\,\beta q_{n}r^{v_{n}}}}{\beta^{2}}\,+\,\frac{r^{2}}{2}\!\right)\!\!\right) \end{aligned}}}  $$

where $q_{n}=\left (C_{L}/C_{N}\right)^{\frac {1}{\alpha _{L}}}, v_{l}=\alpha _{N}/\alpha _{L} $ and

5 $$ \mathcal{A}_{N}=1-\mathcal{A}_{L}  $$

is the probability that the reference BS is connected to a NLOS typical user.

### Antenna gain pattern and directivity

All users and BSs are equipped with directional antennas with sectorized gain pattern as in [22]. The directivity gain at the BS is taken as a constant *M*_*r*_ for all angles in the main lobe, and another constant *m*_*r*_ for the side lobes. Hence, given the beamwidth of the main lobe as *θ*_*r*_, the gain function of the BS at angle *ψ*_*r*_ off the boresight direction can be represented by $G_{M_{r},m_{r},\theta _{r}}(\psi _{r})$. In the same way, the gain function of the user at an angle *ψ*_*t*_ off the boresight direction can be denoted by $G_{M_{t},m_{t},\theta _{t}}(\psi _{t})$, where *M*_*t*_, *m*_*t*_, and *θ*_*t*_ are the user parameters. Here, we consider that based on the estimated channel, the reference BS and the typical user can adjust their beam steering angles to achieve the maximum array gains. As a result, the total directivity gain of the desired signal is *M*_*r*_*M*_*t*_. Furthermore, for the *l*th interference link, we assume that the angle of departure at the interfering user $\psi _{t}^{l}$ and the angle of arrival at the reference BS $\psi _{r}^{l}$ are independently and uniformly distributed in (0,2*π*], which results in a gain of $G_{l}=G_{M_{t},m_{t},\theta _{t}}\left (\psi _{t}^{l}\right)G_{M_{r},m_{r},\theta _{r}}\left (\psi _{r}^{l}\right)$. Hence, the directivity gain in the interference link *G*_*l*_ is a discrete random variable whose probability distribution is given as *a*_*k*_ with probability *b*_*k*_ (*k*∈{1,2,3,4}), where *a*_1_=*M*_*r*_*M*_*t*_, ${b_{1}=\frac {\theta _{r}\theta _{t}}{4\pi ^{2}}}$, *a*_2_=*M*_*r*_*m*_*t*_, $b_{2}=\frac {\theta _{r}}{2\pi }\left (1-\frac {\theta _{t}}{2\pi }\right)$, *a*_3_=*m*_*r*_*M*_*t*_, $b_{3}=\left (1-\frac {\theta _{r}}{2\pi }\right)\frac {\theta _{t}}{2\pi }$, *a*_4_=*m*_*r*_*m*_*t*_, and $b_{4}=\left (1-\frac {\theta _{r}}{2\pi }\right)\left (1-\frac {\theta _{t}}{2\pi }\right)$ [11].

### User fractional power control

We assume that each user utilizes a distance-proportional FPC of the form $\phantom {\dot {i}\!}R^{\alpha _{0}\tau }$, where *τ*∈[0,1] is the power control factor and *α*_0_ is dependent on the FPC assumption. Therefore, as a user moves closer to its associated BS, the transmit power required to achieve the target received signal power decreases. This is an important consideration in power limited devices such as the battery-powered mobile devices. In general, two FPC schemes can be identified for the mmWave cellular network:

#### Pathloss-based FPC

PL-FPC follows the same approach as in LTE, and hence, only the pathloss which is obtained via reference signals is required for its implementation [24]. PL-FPC operates by the compensating the pathloss of the user irrespective of whether its path to its serving BS is LOS or NLOS. Hence, *α*_0_=*α*_*L*_ for LOS user and *α*_0_=*α*_*N*_ for NLOS user.

#### Distance-based FPC

D-FPC is based on the measured distance and always compensate by inverting with the LOS pathloss exponent, i.e., *α*_0_=*α*_*L*_. As a result, in the D-FPC scheme, each user adjusts the transmit power as if the link to its serving BS were LOS, even if in fact it is NLOS. The scheme requires the knowledge of the user-BS distance which can be readily obtained since the location of the BS is known while that of the user can be estimated by using GPS or position reference symbols. Note that with the PL-FPC, the presence of a single NLOS user can result in significant performance degradation, as it will aim to compensate the NLOS pathloss ($\phantom {\dot {i}\!}R^{-\alpha _{N}}, $ where *α*_*N*_≥4) by transmitting high power $\phantom {\dot {i}\!}R^{\alpha _{N}\tau }$ thereby causing significant interference to other users. Such effect is avoided with the D-FPC where the transmit power remains $\phantom {\dot {i}\!}R^{\alpha _{L}\tau }$ with typical *α*_*L*_ value of 2.

Moreover, if *τ*=0 in either scenario, no channel inversion is performed and all users transmit with the same power.

### Small-scale fading

In order to take the significant difference in the small-scale fading experienced by LOS and NLOS links into consideration, we assume independent Nakagami fading for each link. Positive integer values *N*_*L*_ and *N*_*N*_ are assumed as the Nakagami fading parameters for the LOS and NLOS links, respectively, for simplicity. Let *g*_*l*_ be the small-scale fading term on the *l*th link. Then |*g*_*l*_|^2^ is a normalized gamma random variable.

Based on this and the earlier assumptions, the SINR at the reference BS can be expressed as

6 $$ \text{SINR}=\frac{\lvert g_{0}\rvert^{2}M_{r}M_{t}L(R)R^{\alpha_{0}\tau}}{\sigma^{2}+\sum_{z\in \mathcal{Z}}\lvert g_{z}\rvert^{2}G_{z}L(D_{z})R_{z}^{\alpha_{0}\tau}},  $$

where $\lvert g_{0}\rvert ^{2}M_{r}M_{t}L(R)R^{\alpha _{0}\tau }$ is the received power from the typical user at distance *R* from the reference BS, $\mathcal {Z}$ is the set of interfering users, *D*_*z*_ is the distance between an interfering user and the reference BS, *R*_*z*_ is the distance between an interfering user and its serving BS, *G*_*z*_ is the directivity gain, and *σ*^2^ is the noise power.

## SINR coverage probability

The SINR coverage probability *P*_*c*_(*Γ*) is defined as the probability that the received SINR at the reference BS is above a threshold *Γ*, i.e., $P_{c}(\Gamma)=\mathbb {P}(\text {SINR}>\Gamma)$.

### Distribution of *R*_*z*_

**Fig. 2:**
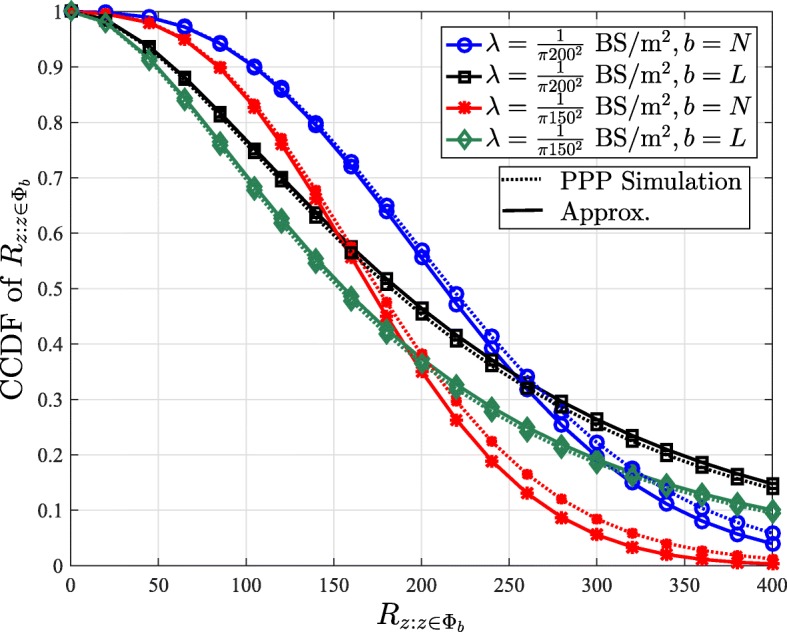
A comparison of the CCDFs of $R_{z:{z\in \Phi _{b}}}$ for the PPP model with their simulation for $\lambda =\frac {1}{\pi 150^{2}}$ and $\frac {1}{\pi 200^{2}}~\mathrm {BS/m^{2}}$

**Fig. 3:**
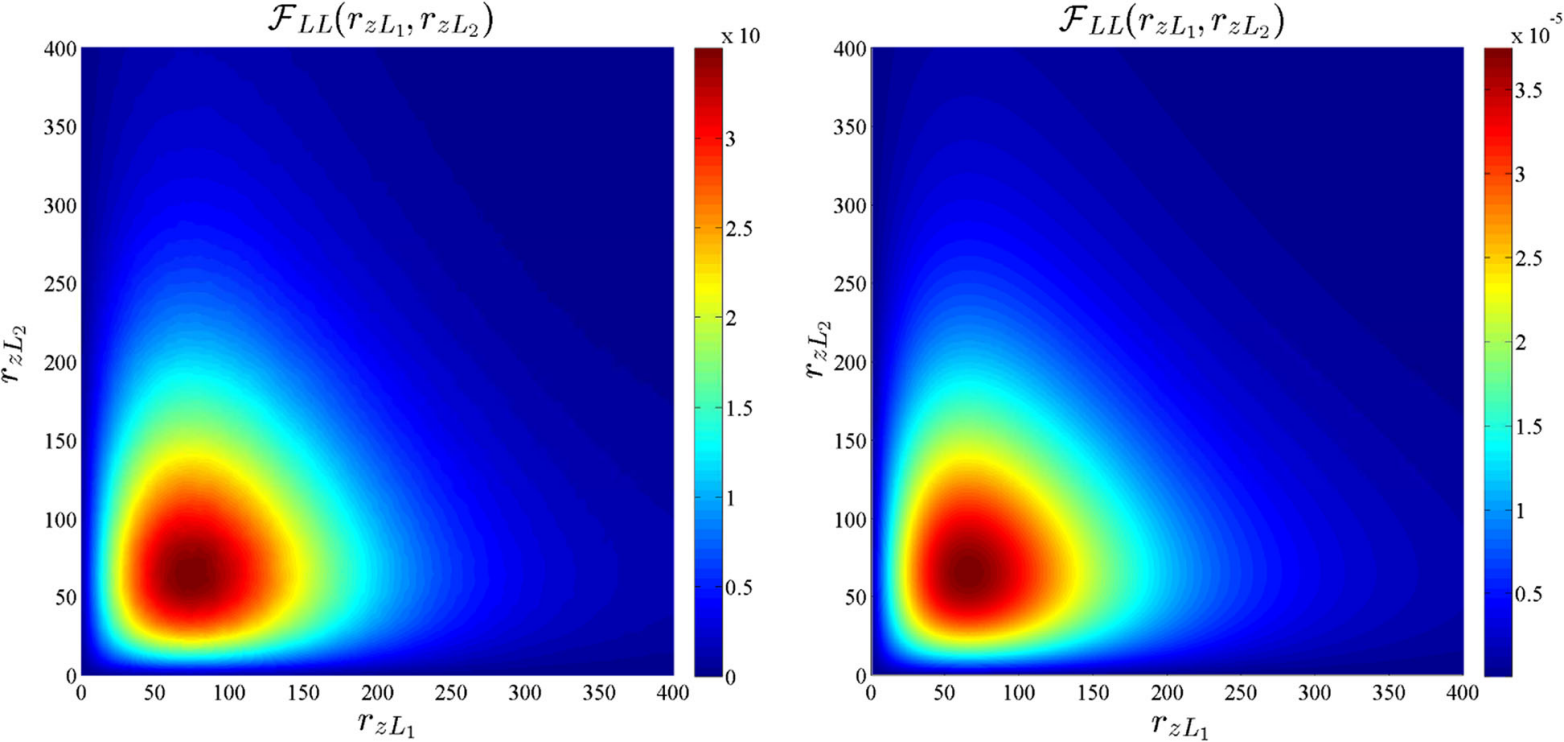
Joint densities of *R*_*zL*1_ and *R*_*zL*2_ for the actual PPP model (left) and the independence assumption (right). *R*_*zL*1_and *R*_*zL*2_ are the distances of LOS users to their respective BSs in two neighboring cells

**Fig. 4:**
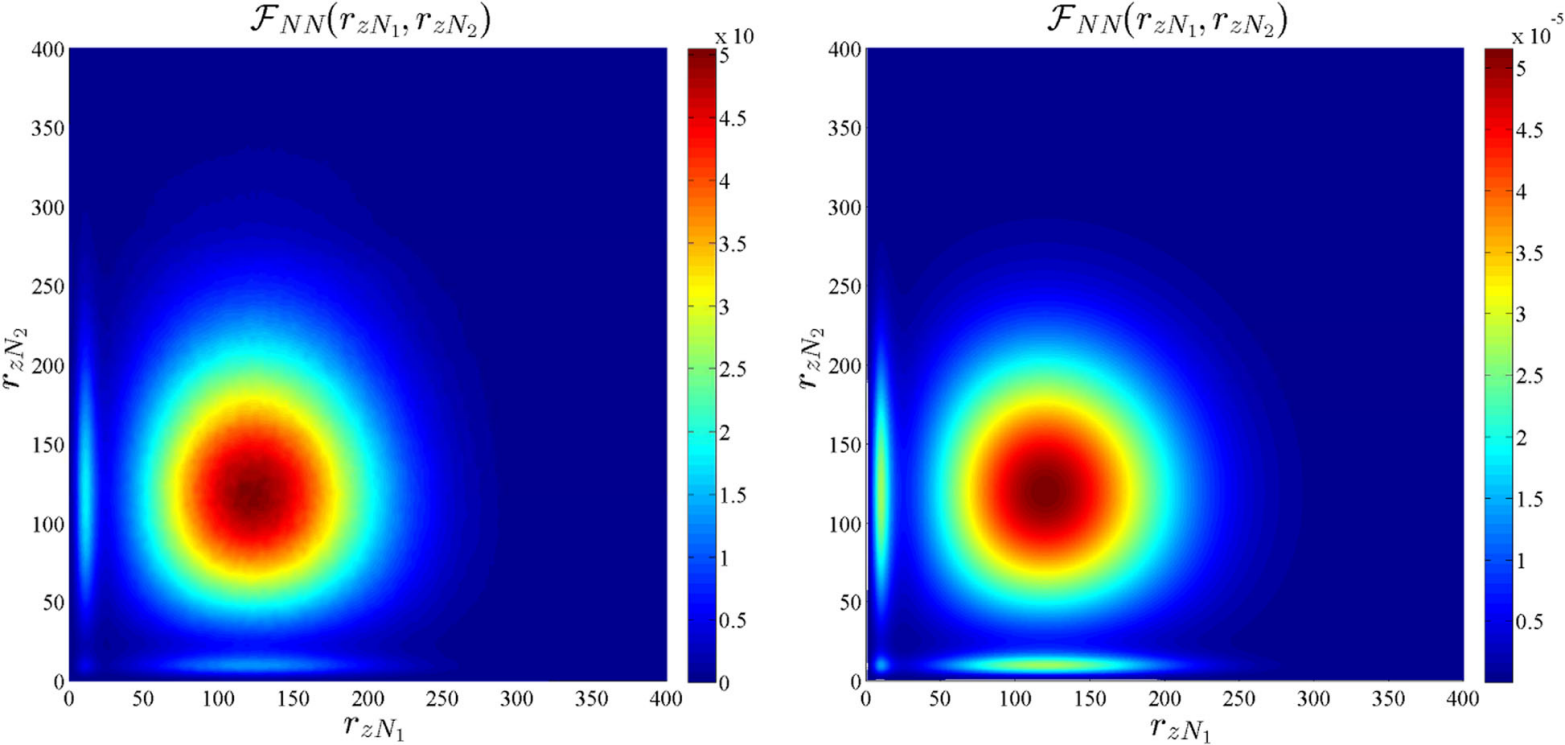
Joint densities of *R*_*zN*1_ and *R*_*zN*2_ for the actual PPP model (left) and the independence assumption (right). *R*_*zN*1_and *R*_*zN*2_ are the distances of LOS users to their respective BSs in two neighboring cells

**Fig. 5:**
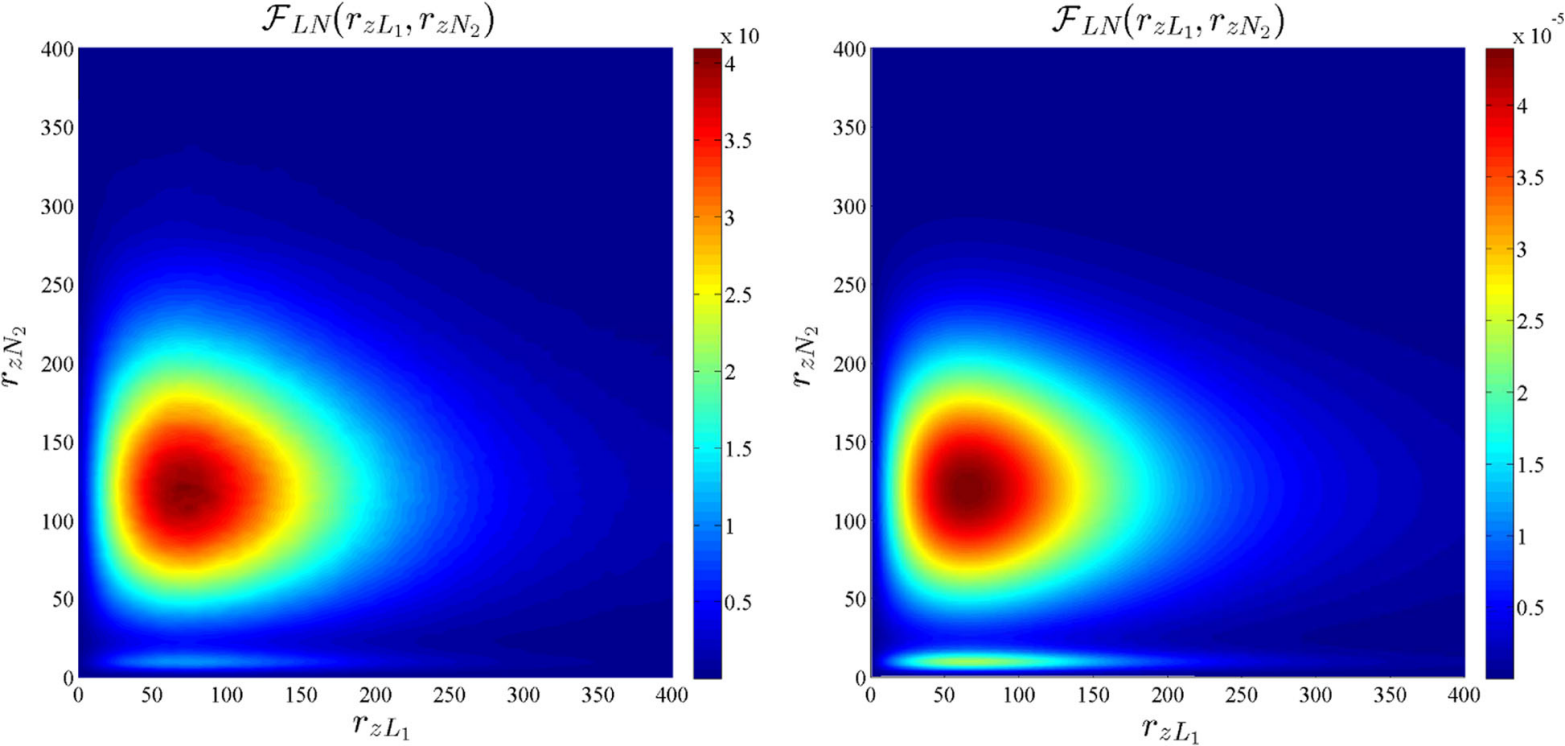
Joint densities of *R*_*zL*1_ and *R*_*zN*2_ for the actual PPP model (left) and the independence assumption (right). *R*_*zL*1_and *R*_*zN*2_ are the distances of LOS users to their respective BSs in two neighboring cells

In order to derive the SINR coverage probability expression, we first derive the distribution of the distance of any interfering user to its serving BS. As mentioned earlier, we represent the set of interfering users by $\mathcal {Z}$, the distance of an interfering user $z\in \mathcal {Z}$ to the BS of interest by *D*_*z*_, and the distance of the interfering user to its serving BS by *R*_*z*_. It should be noted that the random variables $ \{R_{z}\}_{z\in \mathcal {Z}}$ are identically distributed but not independent in general. This dependence is induced by the restriction of having one user served per-BS-per channel, i.e., the coupling of the BS and served user per channel point processes [13, 16, 23]. Here, we demonstrate that this dependence is weak which motivates our independence assumption for $ \{R_{z}\}_{z\in \mathcal {Z}}$. As mentioned in the previous section, each BS have a single user served at any time instant. Therefore, similar to *R*_*L*_ and *R*_*N*_, $R_{z:{z\in \Phi _{b}}}$ for *b*∈{*L*,*N*} can be approximated as the distance of a randomly chosen point in $\mathbb {R}^{2}$, which can either be LOS or NLOS, to the BS that offers the maximum received power, and hence, its distribution can be approximated by

7 $$\begin{array}{@{}rcl@{}} \mathcal{F}_{R_{zL}}(r_{z})&=&\mathcal{F}_{R_{L}}(r_{z})\\ \mathcal{F}_{R_{zN}}(r_{z})&=&\mathcal{F}_{R_{N}}(r_{z}), \end{array} $$

where $\mathcal {F}_{R_{L}}(r_{z})$ and $\mathcal {F}_{R_{N}}(r_{z})$ are defined in (2) and (4), respectively. The CCDF of $R_{z:{z\in \Phi _{b}}}$ for *b*∈{*L*,*N*} is given by $\mathbb {P}\left [R_{z:{z\in \Phi _{b}}}>r_{z}\right ]=\int _{r_{z}}^{\infty }\mathcal {F}_{R_{zb}}(x)~\text {dx}$, which is shown to be a close match for the simulation of the PPP model in Fig. 2. Although Fig. 2 shows that the approximations of the distribution of *R*_*L*_,*R*_*N*_, and $R_{z:{z\in \Phi _{b}}}$, for *b*∈{*L*,*N*}, are accurate, it does not give any insight into the degree of dependence between the random variables $\{R_{z}\}_{z\in \mathcal {Z}}$ which is defined by their joint distribution. Since it is difficult to obtain insights from the complete joint distribution of $\{R_{z}\}_{z\in \mathcal {Z}}$, we focus on a much-simplified scenario of the joint distribution of four random variables *R*_*zL*1_, *R*_*zN*1_, *R*_*zL*2_, and *R*_*zN*2_, which are the distances of LOS and NLOS users to their respective BS in the two neighboring cells. Note that since the dependence is expected to be strongest in neighboring cells, this study illustrates the worst-case scenario. Hence, we numerically compute the joint pdfs $\mathcal {F}_{R_{zL1},R_{zL2}}(r_{zL1},r_{zL2})$, $\mathcal {F}_{R_{zN1},R_{zN2}}(r_{zN1},r_{zN2})$, and $\mathcal {F}_{R_{zL1},R_{zN2}}(r_{zL1},r_{zN2})$ for the actual PPP model and compare them with the joint pdfs under the independence assumptions in Figs. 3, 4, and 5, respectively. The joint pdfs under the independence assumption follow directly from (2) and (4) and are given by:

8 $$\begin{array}{@{}rcl@{}} \mathcal{F}_{R_{zL1},R_{zL2}}(r_{zL1},r_{zL2})&=&\mathcal{F}_{R_{L}}(r_{zL1})\mathcal{F}_{R_{L}}(r_{zL2})\\ \mathcal{F}_{R_{zN1},R_{zN2}}(r_{zN1},r_{zN2})&=&\mathcal{F}_{R_{N}}(r_{zN1})\mathcal{F}_{R_{N}}(r_{zN2}).\end{array} $$

From Figs. 3, 4, and 5, we observe that the pdfs obtained from the actual PPP model and independence assumption are very similar. The correlation coefficient for $\rho _{R_{zL1},R_{zL2}}$, $\rho _{R_{N1},R_{zN2}}$, and $\rho _{R_{zL1},R_{zN2}}$ are numerically computed as 0.00018,0.0467, and − 0.00137, respectively, in the simulation setup. Having validated the independence assumption, we now proceed to derive the SINR coverage probability.

### SINR coverage probability for the case with FPC

The following theorem presents the SINR coverage probability for the PL-FPC. Modifications required for the D-FPC will be presented subsequently.

#### **Theorem 1**

The SINR coverage probability in the uplink of mmWave cellular networks for the case with a PL-FPC can be computed as

9 $$ P_{c}\left(\Gamma\right)=\mathcal{A}_{L} P_{c,L} \left(\Gamma\right)+\mathcal{A}_{N}P_{c,N}\left(\Gamma\right),  $$

where for *b*∈{*L*,*N*}, *P*_*c*,*b*_(*Γ*) is the conditional coverage probability given the reference BS serves a user in *Φ*_*b*_. Moreover, *P*_*c*,*b*_(*Γ*) can be obtained as

$$ {{\begin{aligned} P_{c,L}(\Gamma)\!\approx\!\! \sum_{n=1}^{N_{L}}\!\left(\,-\,1\right)^{n+1}{{N_{L} \choose n}}\!\times\!\!\int_{0}^{\infty} \!\!e^{-{s_{L}}\sigma^{2}\!-\sum_{o\in\{L,N\}}\left(G_{o}(\Gamma,r)+H_{o}(\Gamma,r)\right)}\mathcal{F}_{R_{L}}\!(r)\mathrm{d} r \end{aligned}}} $$

$$ {{\begin{aligned} P_{c,N}(\Gamma)\!\approx\!\!\sum_{n=1}^{N_{N}}\!\left(\,-\,1\right)^{n+1} {N_{N} \choose n}\!\!\times\!\!\int_{0}^{\infty} \!\!e^{-{s_{N}}\sigma^{2}\!-\sum_{o\in\{L,N\}}\left(J_{o}(\Gamma,r)+K_{o}(\Gamma,r)\right)}\mathcal{F}_{R_{N}}\!(r)\mathrm{d} r \end{aligned}}} $$

where

10 $$ {{\begin{aligned} {}G_{o}(\Gamma,r)&=-2\pi\lambda\mathcal{A}_{o}\sum_{k=1}^{4}b_{k}\int_{r}^{\infty} F\left(N_{L},s_{L}a_{k}y^{\alpha_{o}\tau}c^{-\alpha_{L}}\right)\mathrm{c}e^{-\beta c}\mathrm{d} c \\ {} H_{o}(\Gamma,r)&=-2\pi\lambda\mathcal{A}_{o}\!\sum_{k=1}^{4}b_{k}\!\int_{\zeta_{L}(r)}^{\infty}F\!\left(N_{N}\!,s_{L} a_{k}y^{\alpha_{o}\tau}c^{-\alpha_{N}}\right)\left(1\,-\,e^{-\beta c}\right)\!c\mathrm{d} c\\ {}J_{o}(\Gamma,r)&=-2\pi\lambda\mathcal{A}_{o}\sum_{k=1}^{4}b_{k}\int_{\zeta_{N}(r)}^{\infty}\!F\!\left(N_{L},s_{N}a_{k}y^{\alpha_{o}\tau}c^{-\alpha_{L}}\right)e^{-\beta c}c\mathrm{d} c\\ {}K_{o}(\Gamma,r)&=-2\pi\lambda\mathcal{A}_{o\!}\sum_{k=1}^{4}b_{k}\!\int_{r}^{\infty}\!\!F\left(N_{N},s_{N} a_{k}y^{\alpha_{o}\tau}c^{-\alpha_{N}}\right)\left(1\,-\, e^{-\beta c}\right)\!c\mathrm{d} c, \end{aligned}}}  $$

$F(N,x)=1-\int _{0}^{\infty } \mathcal F_{R_{o}}(y)/(1+x)^{N}\mathrm {d}y$, *o*∈{*L*,*N*}, $s_{L}=\frac {\eta _{L}n\Gamma r^{\alpha _{L}(1-\tau)}}{M_{r}M_{t}},s_{N}=\frac {\eta _{N}n\Gamma r^{\alpha _{N}(1-\tau)}}{M_{r}M_{t}}$, ${\zeta _{L}(r)}=\left (\frac {C_{N}}{C_{L}}\right)^{\frac {1}{\alpha _{N}}}\!r^{\frac {\alpha _{L}}{\alpha _{N}}}$, ${\zeta _{N}(r)}=\left (\frac {C_{L}}{C_{N}}\right)^{\frac {1}{\alpha _{L}}}\!r^{\frac {\alpha _{N}}{\alpha _ L}}$, *a*_*k*_ and *b*_*k*_ are antenna directivity parameters defined in Section 2. For $s\in \{L,N\},~\eta _{s}=N_{s}(N_{s}!)^{-\frac {1}{N_{s}}}$ and *N*_*s*_ are the parameters of the Nakagami small-scale fading.

#### *Proof*

See Appendix. □

#### **Corollary 1**

The SINR coverage probability in the uplink of mmWave cellular networks for the case with D-FPC can be computed as in (9) but with *α*_*o*_=*α*_*L*_ and $s_{N}=\frac {\eta _{N}n\Gamma r^{\alpha _{N}-\alpha _{L}\tau }}{M_{r}M_{t}}$ in (10).

#### Noise-limited approximation

Since earlier simulation results in [6, 7] reveals that mmWave networks are more likely to be noise-limited in an urban setting, we also present the noise-limited approximation of the coverage probability. For the noise-limited approximation, $\sigma ^{2}\gg \sum _{z\in \mathcal {Z}}g_{z}L(D_{z})G_{z}R_{z}^{\alpha _{0}\tau }$, the signal-to-noise-ratio (SNR) coverage probability can be expressed from Theorem 1 as

11 $$ {{\begin{aligned} {}P_{c}\left(\Gamma\right)=&\mathcal{A}_{L}\sum_{n=1}^{N_{L}}\left(-1\right)^{n+1}\! {N_{L} \choose n}\int_{0}^{\infty} e^{-\frac{\eta_{L}n\Gamma r^{\alpha_{L}(1-\tau)}}{G_{u}^{\max}G_{b}^{\max}}\frac{\sigma^{2}}{P_{b}^{0}}} \mathcal{F}_{R_{L}}\!(r)\mathrm{d}r\\ &+\!\mathcal{A}_{N}\!\sum_{n=1}^{N_{N}}\!\left(\!-1\!\right)^{n+1}\! {N_{N} \choose n}\!\int_{0}^{\infty} \!e^{-\frac{\eta_{N}n\Gamma r^{\alpha_{N}(1-\tau)}}{G_{u}^{\max}G_{b}^{\max}}\frac{\sigma^{2}}{P_{b}^{0}}}\! \mathcal{F}_{R_{N}}\!(r)\mathrm{d}r \end{aligned}}}  $$

by equating *G*_*o*_(*Γ*,*r*), *H*_*o*_(*Γ*,*r*), *J*_*o*_(*Γ*,*r*), and *K*_*o*_(*Γ*,*r*) to zero.

### Rate and area spectral efficiency

Here, we turn our attention to the distribution of the achievable data rate *Υ* and the area spectral efficiency $\mathcal {S}$ in the uplink of mmWave cellular networks. The achievable data rate can be defined according to [11] as follows

12 $$ \Upsilon=B\ln\left(1+\min\left(\text{SINR},\Gamma_{\max}\right)\right),  $$

where *B* is the bandwidth allocated to the user, *Γ*_max_ is the SINR threshold defined by the order of practical coding and modulation schemes, and the linearity of the radio frequency front-end.

The area spectral efficiency, which is the same as the potential throughput normalized by bandwidth can be obtained from the SINR coverage probability *P*_*c*_(*Γ*) by utilizing the following Lemma.

#### **Lemma 1**

Given the SINR coverage probability *P*_*c*_(*Γ*), the area spectral efficiency of the uplink of a mmWave cellular network can be expressed as

13 $$ \mathcal{S}=\frac{\lambda}{\ln 2}\int_{0}^{\infty}\frac{P_{c}(\Gamma)}{1+\Gamma}\mathrm{d}\Gamma,  $$

which has the unit of bps/Hz/*m*^2^.

#### *Proof*

The proof follows directly from the relationship between the SINR coverage probability and the average ergodic spectral efficiency $\mathcal {R}$, which is given in [25], and the fact that $\mathcal {S}=\lambda \mathcal {R}$. □

## Numerical results and discussion

In this section, we present some numerical results to illustrate our analytical findings in Section 3. We assume that the mmWave network is operated at 28 GHz with 100 MHz allocated to each user. The LOS and NLOS pathloss exponents are taken as *α*_*L*_=2 and *α*_*N*_=4 while the Nakagami fading parameters are *N*_*L*_=3 and *N*_*N*_=2. We consider that LOS probability function *p*(*R*)=*e*^−*βR*^, where 1/*β*=141.4 m. The antenna gain pattern of a BS is assumed to be characterized with *M*_*r*_=10 dB,*m*_*r*_=−10 dB, and *θ*_*r*_=30°, while that of a user is assumed to be characterized with *M*_*t*_=10 dB, *m*_*t*_=−10 dB, and *θ*_*t*_=90°. For comparison purpose, we have also included the conventional stochastic geometry analysis of the uplink channel in [13] that does not differentiate between LOS and NLOS transmission and assumes small-scale Rayleigh fading between the users and BSs (i.e., *N*_*L*_=*N*_*N*_=1). Note that only one pathloss exponent is defined in [13] and is denoted here as *α*=*α*_*N*_. Furthermore, for fairer comparison, we also consider the SINR coverage probability of the UHF network with Nakagami fading parameter *N*=2.

### Accuracy of the analytical framework

**Fig. 6:**
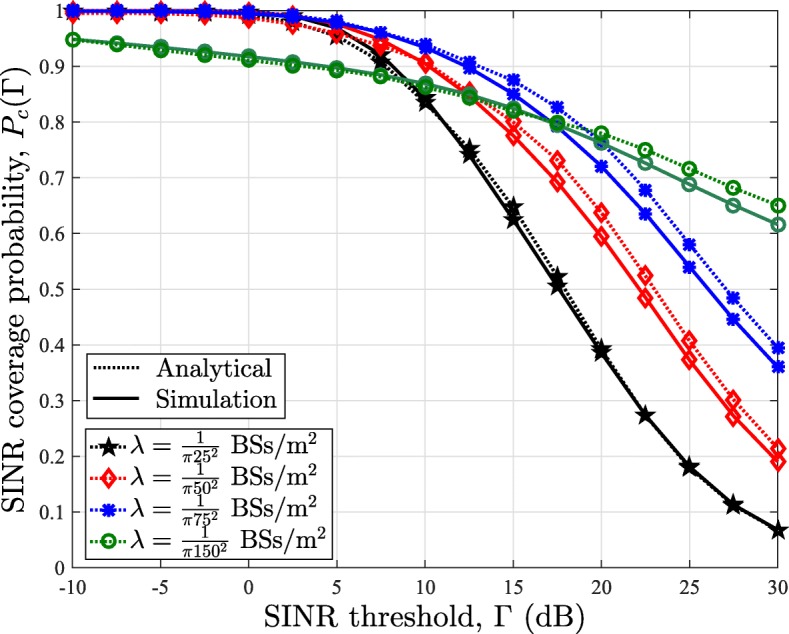
SINR coverage probability in the uplink of mmWave cellular networks

In Fig. 6, we compare the SINR coverage probability obtained via our analytical framework in Theorem 1 with the Monte Carlo simulations for FPC factor *τ*=0. Results in Fig. 6 show that our analytical results in Theorem 1 closely match with the simulation results. Though the gap between derived expressions and simulation results stays small for all tested scenarios, this gap becomes negligible as the density of BS grows. As future mmWave networks are expected to have high BS density, the derived expressions provide a highly accurate method to estimate uplink coverage probability for mmWave networks. Note that the analytical results are based on the independence assumption, and hence, the results in Fig. 6 further validates the accuracy of the independence assumption presented earlier in Figs. 3, 4, and 5.

### D-FPC vs PL-FPC

**Fig. 7:**
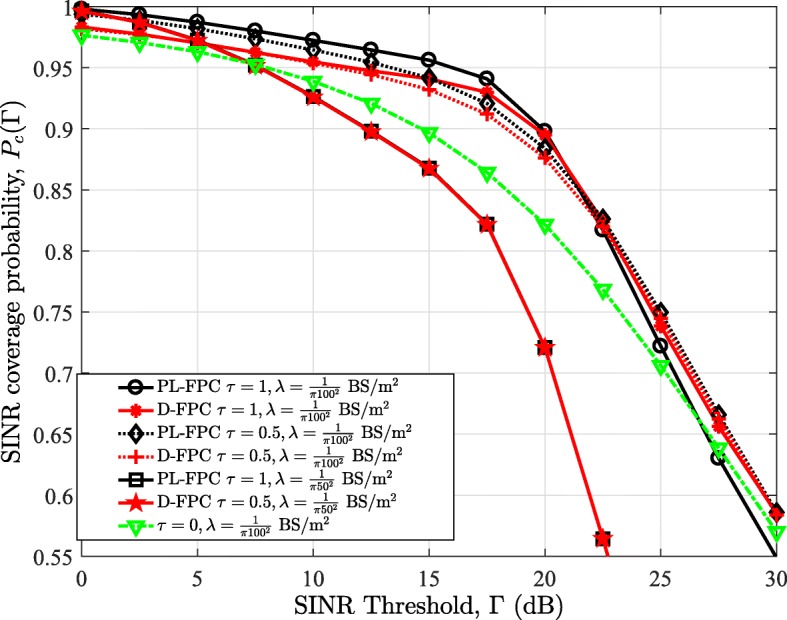
Comparison of the SINR coverage probability based on the PL-FPC and the D-FPC in the uplink of mmWave cellular networks

Figure 7 compares the performance of the D-FPC and PL-FPC schemes for FPC factors *τ*=0.5 and 1, and BS densities $\frac {1}{\pi 50^{2}}$ and $\frac {1}{\pi 100^{2}}~\mathrm {BS/m^{2}}$. Both power control schemes are also benchmarked with the case without power control, i.e., *τ*=0. The results in Fig. 7 show that the D-FPC scheme has greater coverage at high SINR thresholds, for $\lambda =\frac {1}{\pi 100^{2}}\mathrm {BS/m^{2}}$ and full FPC, i.e., *τ*=1, compared with the PL-FPC scheme. This is due to the fact that more users suffer from higher interference as a result of the NLOS users’ channel inversion in the PL-FPC scheme; hence, a higher proportion of users are with lower SINR in PL-FPC. The coverage margin between the two FPC methods, however, reduces as the FPC factor is reduced to 0.5. Furthermore, as the BS density is increased, to $\frac {1}{\pi 50^{2}}\mathrm {BS/m^{2}}$, the D-FPC and PL-FPC converge. This is due to the fact that increasing the BS density increases the tendency of having LOS association, and hence, PL-FPC converges to D-FPC when $\mathcal {A}_{L}\rightarrow 1$.

Note that in our framework, the value of the FPC factor *τ*∈[ 0,1] can be adjusted for both the D-FPC and PL-FPC schemes. Surely by fixing the FPC factor for the D-FPC scheme, we can adjust the FPC factor for users with NLOS links in the PL-FPC scheme to achieve the same power and coverage performance as the D-FPC scheme. However, this implies having different FPC factor for LOS and NLOS users in the PL-FPC scheme analysis. Meanwhile, our analysis has not considered this since we have assumed that the network selects utilizes a global power control factor.

### Effect of BS density

**Fig. 8:**
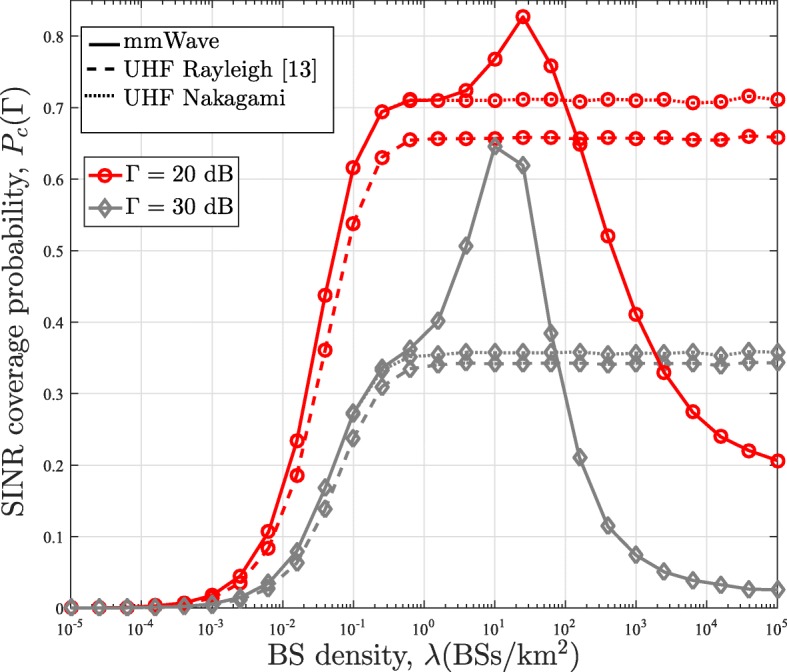
Comparison of the SINR coverage probability of the mmWave and UHF networks in the uplink channel with the power control factor *τ*=0

**Fig. 9:**
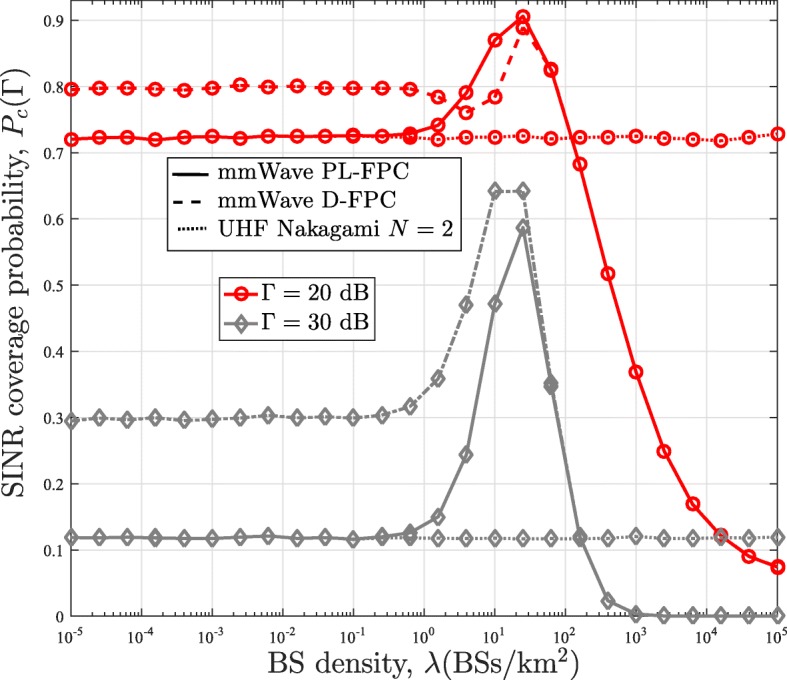
Comparison of the SINR coverage probability of the mmWave and UHF networks in the uplink channel with the power control factor *τ*=1

In Figs. 8 and 9, we plot the SINR coverage distribution obtained from our analytical framework as a function of the BS density for the case with no power control, i.e., *τ*=0, and full power control (PL-FPC and D-FPC), i.e., *τ*=1, respectively. The plots in Fig. 8 are also benchmarked with the results obtained from the conventional stochastic geometry analysis for the uplink channel in [13]. For the case without power control (*τ*=0) in Fig. 8, the coverage probability performance obtained from the conventional stochastic geometry analysis in [13] initially increases with the BS density. This is due to the fact that having more BSs leads to improved coverage in the noise-limited network (i.e., eliminates coverage hole). When *λ* is large enough (e.g., *λ*>10^−1^ BSs/k*m*^2^), the SINR coverage probability becomes independent of the BS density as the network becomes interference limited. The simple pathloss model is responsible for this behavior as the increased interference is being counterbalanced by the increase in the signal power as *λ* increases in the interference limited network. In the mmWave framework, the same observation, which follows the conventional analysis, is experienced in the noised limited region. However, when the mmWave network becomes denser than a certain threshold, the coverage probability starts decreasing. The reason behind this is that NLOS interference paths are converted to LOS interference paths.

For the case with full power control in Fig. 9, increasing the BS density does not have any impact on the SINR coverage probability obtained from the conventional framework. On the contrary, the coverage probability of the mmWave framework with PL-FPC remains the same with increasing BS density until a threshold where it starts rising to its peak and then decreases afterward. Implementing full power control for the conventional framework implies that the transmit power of all users reduces as the BS density increases, and hence, the SINR coverage probability remains unaffected. Whereas in the mmWave network, NLOS paths convert to LOS paths as the BS density increases. This results in the reduction of the users transmit power, which causes an initial increase in the SINR coverage probability. However, similar to mmWave network with no power control, the likelihood of having an LOS interferer also increases. This consequently results in the reduction in the SINR coverage probability as its effect eventually predominated that of the transmit power reduction. Regarding the D-FPC, it outperforms the PL-FPC at low BS density and converges to the PL-FPC at high BS density. This convergence is expected since all path becomes LOS at very high BS density. Furthermore, for the UHF network with Nakagami fading, it can be observed that its SINR coverage probability converges to that of mmWave with when there is no power control and *λ*<10^−0.2^BS/k*m*^2^. A similar observation can be seen for the PL-FPC with full powercontrol.

**Fig. 10:**
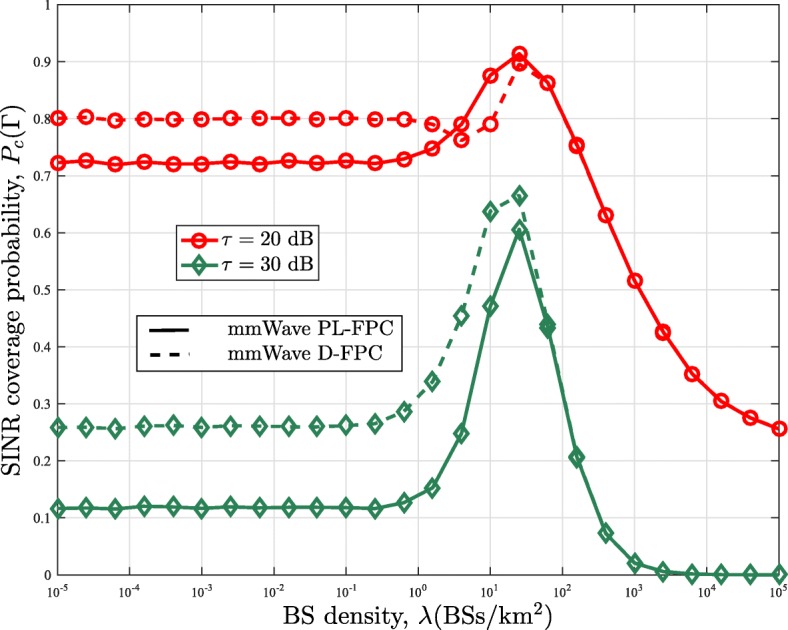
SINR coverage probability with the power control factor *τ*=1 and propagation environment with *α*_*L*_=2.5 and *α*_*N*_=4

In Fig. 10, we present results for other propagation environment. In particular, we considered the NLOS pathloss exponent *α*_*N*_=4 and LOS pathloss exponent *α*_*L*_=2.5. It can be seen that the earlier observations equally hold for this propagation environment.

### Noise-limited approximation

**Fig. 11:**
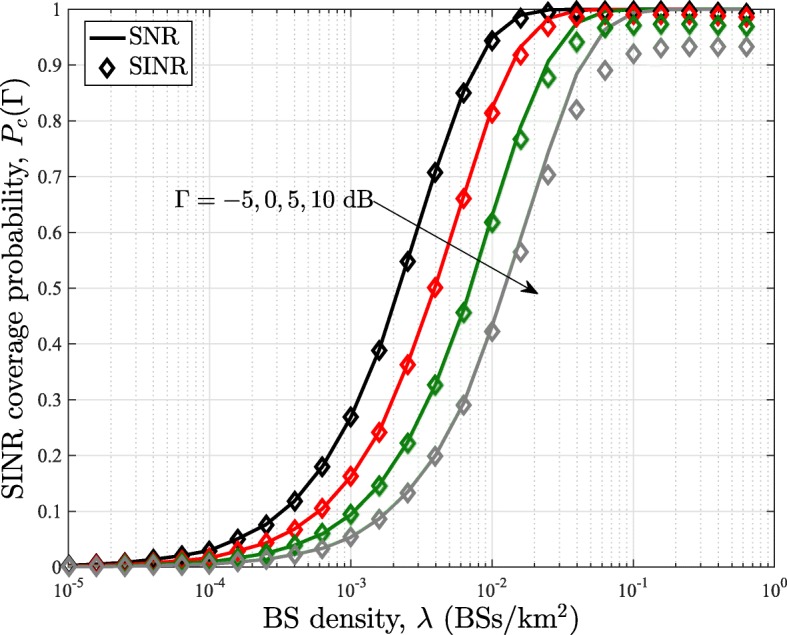
Comparison of SINR and SNR coverage probability in the uplink of mmWave cellular networks

In Fig. 11, we show the results based on the SNR coverage probability, which has been obtained from the noise-limited approximation of the SINR coverage probability in (11), and for *τ*=0. It can be observed that the SNR coverage probability tracks the SINR coverage probability for a threshold *Γ*<5 dB and BS density *λ*<10^−1.8^ BSs/k*m*^2^. However, for very large BS densities, the interference dominates and a gap can be seen between the SINR and SNR coverage plots.

### Area spectral efficiency

**Fig. 12:**
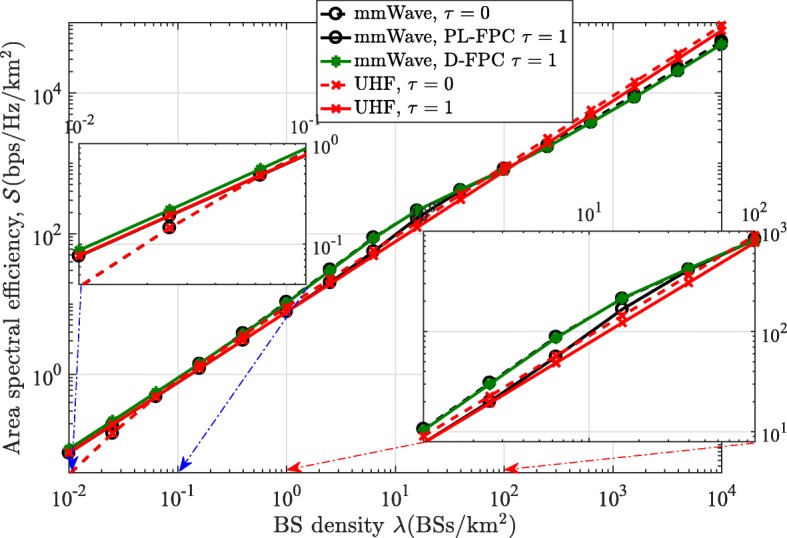
Comparison of the area spectral efficiency of the mmWave and UHF networks in the uplink channel for *τ*=0 and *τ*=1

Figure 12 gives the area spectral efficiency of both mmWave and UHF networks as a function of BS density *λ*, for FPC *τ*=0 and 1. As it can be observed, the area spectral efficiency of the UHF network with *τ*=0 increases invariably linearly with *λ*, when *λ* is large enough, e.g., *λ*≥10^−1^ BS/k*m*^2^. Whereas, for *τ*=1, its area spectral efficiency increases linearly without a restriction on *λ*. This can be implied from the results in Figs. 8 and 9 where the SINR coverage probability of the UHF model becomes constant with increased *λ*, i.e., *λ*≥10^−1^ BS/k*m*^2^ for *τ*=0, while the SINR coverage probability is constant over all *λ* values for *τ*=1. On the other hand, the mmWave network experiences a slow growth region between *λ*=10^1^ BS/k*m*^2^ and *λ*=10^3^ BS/k*m*^2^, which is due to the sharp decrease in the SINR coverage probability at that region. The results also show that the area spectral efficiency of the mmWave network with D-FPC converges to that with PL-FPC as the BSs become very dense (*λ*≥10^2^ BS/k*m*^2^). Furthermore, the area spectral efficiency of the mmWave network (with PL-FPC) converges to that of the UHF model when *λ*≤10^−0.2^ BS/k*m*^2^ and *λ*≤10^−1.4^ BS/k*m*^2^, for *τ*=1 and *τ*=0, respectively. A similar trend in SINR coverage probability and area spectral efficiency performances have been observed for the downlink channel of mmWave networks in [26].

## Conclusions

In this paper, we have presented a stochastic geometry framework to analyze the coverage in the uplink of millimeter wave cellular networks. The framework takes the effect of blockage into consideration by utilizing a distance-dependent line-of-sight probability function and modeling the location of LOS and non-LOS users as two independent non-homogeneous Poisson point processes. The proposed model takes into account per-user fractional power control, which couples the transmission of users due to location-dependent channel inversion. The numerical results show that there exists a density of mmWave base stations that maximizes the SINR coverage probability

## Appendix

### Proof of Theorem 1

Given that the link between the desired (typical) user and the reference BS is LOS, the conditional coverage probability can be computed as

14 $$\begin{array}{@{}rcl@{}} P_{c,L}(\Gamma)&=&\int_{0}^{\infty}\mathbb{P}[\text{SINR}\!>\!\Gamma]\mathcal{F}_{R_{L}}(r)\mathrm d r\\ &=&\int_{0}^{\infty}\mathbb{P}\left[\lvert g_{0}\rvert^{2}>r^{\alpha_{L}(1-\tau)}\Gamma Q/\!(M_{r}M_{t})\right]\mathcal{F}_{R_{L}}(r)\mathrm{d} r \end{array} $$

where *Q*=*I*_*LL*_+*I*_*LN*_+*I*_*NL*_+*I*_*NN*+_*σ*^2^,

15 $$\begin{array}{*{20}l} I_{LL}&=\sum_{l:X_{l}\in\Phi_{L}\cap\bar {\mathcal{B}}(0,r)\cap L}\lvert g_{l}\rvert^{2}G_{l}D_{l}^{-\alpha_{L}}R_{l}^{\alpha_{L}\tau},\\ I_{LN}&=\sum_{l:X_{l}\in\Phi_{L}\cap\bar {\mathcal{B}}(0,r)\cap N} \lvert g_{l}\rvert^{2}G_{l}D_{l}^{-\alpha_{L}}R_{l}^{\alpha_{N}\tau},\\ I_{NL}&=\sum_{l:X_{l}\in\Phi_{N}\cap\bar {\mathcal{B}}(0,\zeta_{L}(r))\cap L} \lvert g_{l}\rvert^{2}G_{l}D_{l}^{-\alpha_{N}}R_{l}^{\alpha_{L}\tau}~\text{and}\\ I_{NN}&=\sum_{l:X_{l}\in\Phi_{N}\cap\bar {\mathcal{B}}(0,\zeta_{L}(r))\cap N} \lvert g_{l}\rvert^{2}G_{l}D_{l}^{-\alpha_{N}}R_{l}^{\alpha_{N}\tau} \end{array} $$

are the interferences experienced at the reference BS from the LOS users with LOS links to their serving BSs, LOS users with NLOS links to their serving BSs, NLOS users with LOS links to their serving BSs, and NLOS users with NLOS links to their serving BSs, respectively; $\mathcal {B}(0,r)$ denotes a disc of radius *r* centered at the origin and $\bar {\mathcal {B}}(0,r)$ denotes outside $\mathcal {B}(0,r)$. The CCDF of the SINR at distance *r* from the reference BS is

16 $$ {\begin{aligned} \mathbb{P}\Big[\lvert g_{0}\rvert^{2}&>r^{\alpha_{L}(1-\tau)}\Gamma Q/(M_{r}M_{t})\Big]\\&\overset{(a)}\approx1-\mathbb{E}_{\Phi}\left[\left(1-e^{\left({-\eta_{L}r^{\alpha_{L}(1-\tau)}\Gamma Q}/({M_{r}M_{t}})\right)}\right)^{N_{L}}\right]\\ &\overset{(b)}=\sum_{n=1}^{N_{L}}\!\!\left(-\!1\right)^{n+1}\!\! {N_{L} \choose n}\mathbb{E}_{\Phi}\!\left[\!e^{\left({-\eta_{L} nr^{\alpha_{L}(1-\tau)}\Gamma Q}/({M_{r}M_{t}})\right)}\!\!\right]\\ &\overset{(c)}=\sum_{n=1}^{N_{L}}\!\left(-1\right)^{n+1}\! {N_{L} \choose n}\!\exp{\left(-s_{L}\sigma^{2}\!\right)\!}\prod_{i,j\in{L,N}}\mathcal{L}_{I_{i,j}}(s_{L}) \end{aligned}}  $$

where $s_{L}= \frac {\eta _{L}n r^{\alpha _{L}(1-\tau)}\Gamma }{M_{r}M_{t}},~\eta _{L}=N_{L}(N_{L}!)^{-\frac {1}{N_{L}}}$, (*a*) follow from the fact that |*g*_0_|^2^ is a normalized gamma random variable with parameter *N*_*L*_ and the fact that for a constant *γ*>0, the probability $\mathbb {P}(\lvert g_{0}\rvert ^{2}<\gamma)$ is tightly upper bounded by $\left [1-\exp \left (-\gamma N_{L}\left (N_{L}!\right)^{-\frac {1}{N_{L}}}\right)\right ]^{N_{L}}$ [27]. (*b*) follows from the binomial theorem and the earlier assumption that *N*_*L*_ is a positive integer, and (*c*) follows from the definition of Laplace transform of interference $\mathcal {L}_{I_{i,j}}(s_{L})=\mathbb {E}_{I_{i,j}}\left [e^{-s_{L}I_{i,j}}\right ]$. To complete the derivation, stochastic geometry concepts can be applied to derive the expression for $\mathcal {L}_{I_{LL}}(s_{L})$ in (16) as

17 $$ {{\begin{aligned} {}\mathcal{L}&_{I_{LL}}(s_{L})=\mathbb{E}_{I_{LL}}[e^{-s_{L} I_{LL}}]\\ &{}=\mathbb{E}_{\Phi_{L}}\left[\exp{\left\{-s_{L}\sum_{z:X_{z}\in\Phi_{L}\cap\bar {\mathcal{B}}(0,r)\cap L}\lvert g_{z}\rvert^{2}G_{z}D_{z}^{-\alpha_{L}}R_{z}^{\alpha_{L}\tau}\right\}}\right]\\ &{}=\mathbb{E}_{R_{z},G_{z},D_{z},g_{z}}\left[ \prod_{z:X_{z}\in\Phi_{L}\cap\bar {\mathcal{B}}(0,r)\cap L}\exp\left\{-s_{L} \lvert g_{z}\rvert^{2}G_{z}D_{z}^{-\alpha_{L}}R_{z}^{\alpha_{L}\tau}\right\}\right]\\ &{}\overset{(d)}=\exp{\left(\,-\,2\pi\lambda\mathcal{A}_{L}\!\sum_{k=1}^{4}\!b_{k}\!\!\int_{r}^{\infty} \!\!\!e^{-\beta c}\left(1\!\,-\,\mathbb{E}_{R_{z},g}\!\left[\exp\left\{\,-\,s_{L} gc^{-\alpha_{L}}R_{z}^{\alpha_{L}\tau}\!\right\}\right]\right)\!c\mathrm dc\!\right)}\\ &{}\overset{(e)}=\exp{\!\left(\!-2\pi\lambda\mathcal{A}_{L}\sum_{k=1}^{4}\!b_{k}\!\int_{r}^{\infty} \!\!e^{-\beta c}\left(\!1\!\,-\,\!\mathbb{E}_{R_{z}}\!\!\left[\!\frac{1}{1\,+\,s_{L}a_{k}c^{-\alpha_{L}}R_{z}^{\alpha_{L}\tau}}\!\right]^{N_{L}}\!\right)\!c\mathrm dc\!\right)} \\ &{}\overset{(f)}=\prod_{k=1}^{4}\!\!\exp\!\!{\left(\,-\,2\pi\lambda\mathcal{A}_{L}b_{k}\!\!\int_{r}^{\infty} \!\!e^{-\beta\! c\!}\left(1\,-\,\!\int_{0}^{\infty}\!\!\frac{\mathcal F_{R_{L}}(y)}{(1\,+\,s_{L}a_{k}c^{-\alpha_{L}}y^{\alpha_{L}\tau})^{N_{L}}}\mathrm dy\!\!\right)\!c\mathrm dc\!\!\right)} \\ &{}=e^{-G_{L}(\Gamma,r)}, \end{aligned}}}  $$

where *g* in (*d*) is a normalized gamma variable with parameter *N*_*L*_, *a*_*k*_ and *b*_*k*_ are defined in earlier in Section 2, (*d*) follows from the probability generating functional of the PPP [12], and the independence of the interference link directivity gain *G*_*z*_ with probability distribution *G*_*z*_=*a*_*k*_with probability *b*_*k*_. Furthermore, *λ* is thinned by $\mathcal {A}_{L}$ to capture *R*_*z*_ that are LOS to their serving BS. (*e*) follows from computing the moment generating function of a gamma random variable *g*, and (*f*) follows from the independence of $\{R_{z}\}_{z\in \mathcal Z}$ which has been validated earlier in Section 3.1 and the fact that the interfering users are in LOS to their serving BS. The computation for $\mathcal L_{I_{LN}}(s_{L})$ which denotes the Laplace transform of LOS interfering links with NLOS links to their serving BS can be obtained by following the same process such that,

18 $$ {{\begin{aligned} {}\mathcal L&_{I_{LN}}(s_{L})= \mathbb{E}_{I_{LN}}[e^{-s_{L} I_{LN}}]\\ {}&=\prod_{k=1}^{4}\!\exp\!{\left(\!-2\pi\lambda\mathcal{A}_{N}b_{k}\int_{r}^{\infty} \!\!e^{-\beta c}\left(1\,-\,\int_{0}^{\infty}\!\frac{\mathcal F_{R_{N}}(y)}{\!\!(1\,+\,s_{L}a_{k}c^{-\alpha_{L}}y^{\alpha_{N}\tau})^{N_{L}}}\mathrm \!dy\right)c\mathrm dc\right)} \\ {}&=e^{-G_{N}(\Gamma,r)}. \end{aligned}}}  $$

Similarly, for the NLOS interfering links which are in LOS to their serving BS, $\mathcal L_{I_{NL}}(s_{L})$ in (16) can be computed as

19 $$ {{\begin{aligned} {}\mathcal L&_{I_{NL}}(s_{l})=\mathbb{E}_{I_{NL}}[e^{-s_{L} I_{NL}}]\\ {}&=\mathbb{E}_{\Phi_{N} }\left[\exp\left\{-s_{L} \sum_{z:X_{z}\in\Phi_{L}\cap\bar{\mathcal B} (0,\zeta_{L}(r))\cap L}\lvert g_{z}\rvert^{2}G_{z}D_{z}^{-\alpha_{N}}R_{z}^{\alpha_{L}\tau}\right\}\right]\\ {}&=\prod_{k=1}^{4}\exp\!{\left(\!\!-2\pi\lambda\mathcal{A}_{L}b_{k}\!\!\int_{\zeta_{L}(r)}^{\infty}\!\! (1\,-\,e^{-\beta c})\!\left(\!\!1\,-\,\!\int_{0}^{\infty}\!\!\frac{\mathcal F_{R_{L}}(y)}{(1\,+\,s_{L}a_{k}c^{-\alpha_{N}}y^{\alpha_{L}\tau})^{N_{N}}}\mathrm dy\!\right)\!c\mathrm dc\!\right)}\\ {}&=e^{-H_{L}(\Gamma,r)}. \end{aligned}}}  $$

Furthermore, for NLOS interfering links which are NLOS to their serving BS, $\mathcal L_{I_{NN}}(s_{L})$ in (16) can be computed as

20 $$ {{\begin{aligned} {}\mathcal L&_{I_{NN}}(s_{L})= \mathbb{E}_{I_{NN}}[e^{-s_{L} I_{NN}}]\\ {}&=\prod_{k=1}^{4}\exp\!{\left(\!\!\!-2\pi\!\lambda\!\mathcal{A}_{N}b_{k}\!\!\int_{\zeta_{L}\!(\!r\!)}^{\infty} (\!1-\!e^{-\beta c})\!\!\left(\!\!1-\!\int_{0}^{\infty}\!\!\!\frac{\mathcal F_{R_{N}}(y)}{(1\,+\,s_{L}a_{k}c^{-\alpha_{N}}\!y^{\alpha_{N}\tau})^{N_{N}}}\mathrm dy\!\right)\!c\mathrm dc\!\right)}\\ {}&=e^{-H_{N}(\Gamma,r)} \end{aligned}}}  $$

Hence, we obtain (10) by substituting for $\mathcal L_{I_{i,j}}(s_{L})$ in (16), which is further substituted into (14).

Given that the link between the desired user and the reference BS is NLOS, we can also compute the conditional probability *P*_*c*,*N*_(*Γ*) by following the same approach as that of *P*_*c*,*L*_(*Γ*). Thus, we omit the detailed proof of (10) here.

Consequently, from the law of total probability, it follows that $P_{c}\left (\Gamma \right)=\mathcal A_{L} P_{c,L} \left (\Gamma \right)+\mathcal A_{N}P_{c,N}\left (\Gamma \right)$.
